# Prevalence and Genetic Diversity of *Listeria monocytogenes* Isolated From Retail Pork in Wuhan, China

**DOI:** 10.3389/fmicb.2021.620482

**Published:** 2021-03-09

**Authors:** Yiqian Wang, Qiang Ji, Shaowen Li, Mei Liu

**Affiliations:** ^1^College of Veterinary Medicine, Huazhong Agricultural University, Wuhan, China; ^2^College of Life Science and Technology, Huazhong Agricultural University, Wuhan, China

**Keywords:** *Listeria monocytogenes*, genetic diversity, pathogenic island, antibiotic-resistant genes, ST619, cgMLST, SNP, CRISPR-Cas system

## Abstract

*Listeria monocytogenes* is a ubiquitous bacteria and causative agent of zoonotic listeriosis with high mortality. The consumption of contaminated animal-derived foods has been linked with both epidemic and sporadic listeriosis. In this work, a total of 64 *L. monocytogenes* isolates from 259 pork samples sold in 11 supermarket chains were identified and characterized by comparative whole-genome analysis. All isolates were delineated into eight clonal complexes (CCs), namely CC2, CC8, CC9, CC11, CC155, CC121, CC204, and CC619, spanning two lineages (I and II) and carrying 3–5 antibiotic-resistant genes (*fosX*, *lnu*, *mprF*, *tetM*, and *dhfR*). It is noted that *Listeria* pathogenicity island (LIPI)-1, LIPI-3, and LIPI-4 were distributed in all ST619 isolates from two supermarket chains that were closely related with clinical isolates (<40 SNP). Some of the isolates from different supermarket chains with 0 SNP difference indicated a common pork supply source. Notably, 57.81% of the strains carried types IB, IIA, or IIIB CRISPR-Cas system, CC121 isolates carried both types IB and IIA CRISPR-Cas systems, Cas proteins of CC155 isolates located between two CRISPR loci, each CC has unique organization of Cas proteins as well as CRISPR loci. CRISPR-Cas system-based subtyping improved discrimination of pork-derived *L. monocytogenes* isolates. Comparisons at the genome level contributed to understand the genetic diversities and variations among the isolates and provided insights into the genetic makeup and relatedness of these pathogens.

## Introduction

*Listeria monocytogenes* is a Gram-positive, facultative intracellular pathogen that is ubiquitously distributed in the environment (Smith et al., 2019). A wide variety of animal species can be infected by *L. monocytogenes*, including mammals, birds, fish and crustaceans (OIE terrestrial manual 2018. Chapter 3.9.6 – *L. monocytogenes*). Pigs rarely develop listeriosis when exposed to *L. monocytogenes* contaminated feed (OIE terrestrial manual 2018. Chapter 3.9.6 – *L. monocytogenes*), while they may become carriers of *L. monocytogenes*, and the pathogen is transmissible to humans through the pig and pork production chain to cause human listeriosis (Félix et al., 2018). Listeriosis outbreaks are reported from time to time worldwide. In South Africa, an outbreak claimed the lives of 193 patients caused by *Listeria* contaminated pork sausage in 2017 (Thomas et al., 2020). In France, an outbreak (32 cases, 10 deaths) occurred because of *L. monocytogenes* contamination in pork product in 1999–2000 (Matle et al., 2020). In United States, CDC reported an outbreak of *Listeria* infection linked to pork products in 2018^1^. The contamination and transmission of *L. monocytogenes* along pork production chain poses serious threats to public health and food safety, it has become a concern for countries around the world.

Understanding the prevalence of *L. monocytogenes* in pork can provide fundamental data to evaluate the risk of *Listeria* infecting human. The investigations of *L. monocytogenes* contamination in pork have been carried out in most countries. In the European Union in 2018, *L. monocytogenes* in pig meat was detected in 1.3% of the 24,814 units tested, and in 0.8% (11 out of 1,365) of the tested samples at retail (EFSA and ECDC, 2019). In South Africa, the occurrence of *L. monocytogenes* was higher in pork (13.3%; 18/135) in 2014–2016 (Matle et al., 2019). In Mexico, *L. monocytogenes* was present in 16/79 (20.3%) pork samples collected from two slaughterhouses from 2014 to 2015 in southern Sonora (Figueroa-López et al., 2019). In China, *L. monocytogenes* contaminations in retail raw pork (11.68–30%), chilled pork (11–55%), and RTE pork (4.61–13.5%) have been reported in recent years (Yin et al., 2015; Li et al., 2018; Chen et al., 2019; Zhang et al., 2019b). Pork is the most frequently consumed meat in China, the main consumption habit is to take cooked pork. Whereas, with the change of lifestyle, the frequency of consumption RTE food is increasing, the contamination in RTE pork (4.61–13.5%) suggested that cross-contamination in the process of cooked food increased the infection risk to human. However, the system of surveillance is not clearly described at food and clinical levels.

*Listeria monocytogenes* is a highly heterogeneous species; its population structure is divided into 14 serotypes (Yin et al., 2019) and four phylogenetic lineages (I, II, III, and IV) (Orsi et al., 2011) that have been classified into multiple clonal complexes (CCs) on the basis of multilocus sequence typing (MLST) (Ragon et al., 2008). In the western countries, CCs (1–6) of lineage I are the major types responsible for human epidemics. Further, CC8, CC9, CC121, and CC155 of lineage II are the main causative CCs of human listeriosis; however, their frequency of being reported is lower than that of lineage I isolates, which mainly cause sporadic listeriosis (Chenal-Francisque et al., 2011; Chen et al., 2016; Maury et al., 2016). In China, listeriosis epidemics are different from those in other countries; outbreaks are rarely reported, and sporadic cases are more common, which can be attributed to certain relationships among the predominant clones CC87, CC8, and CC9 in food (Li et al., 2019; Yin et al., 2019; Zhang et al., 2019a). Particularly, the previous studies reported the circulation of typical *L. monocytogenes* CC87 clone in food and clinical disease (Wang et al., 2019; Yin et al., 2020). *L. monocytogenes* is a relatively conservative and constantly varying species. Dynamic epidemic investigations are particularly necessary to understand the genetic diversities and structures of related virulent genes, antibiotic-resistant genes, and horizontal moving elements as well as CRISPR-Cas system.

Wuhan city in Hubei province has 14 million residents who are mainly of the Han ethnicity, and pork meat is the most often consumed animal product in their diet. Although people have gradually been paying more attention to food safety, epidemic investigation data on pork meat in Wuhan city is still limited. In this study, the *L. monocytogenes* contamination status of pork meat in 53 supermarkets belonging to 11 supermarket chains was investigated. The genomic analyses for genetic diversity, virulence, antibiotic resistance, and CRISPR-Cas systems of *L. monocytogenes* isolates were performed. Our research is expected to be beneficial for understanding the prevalence and characterization of *Listeria* contamination in retail pork in China, which is necessary for effectively solving problems related to animal-derived foods and ensuring safety from farm to table.

## Materials and Methods

### Isolation

**FIGURE 1:**
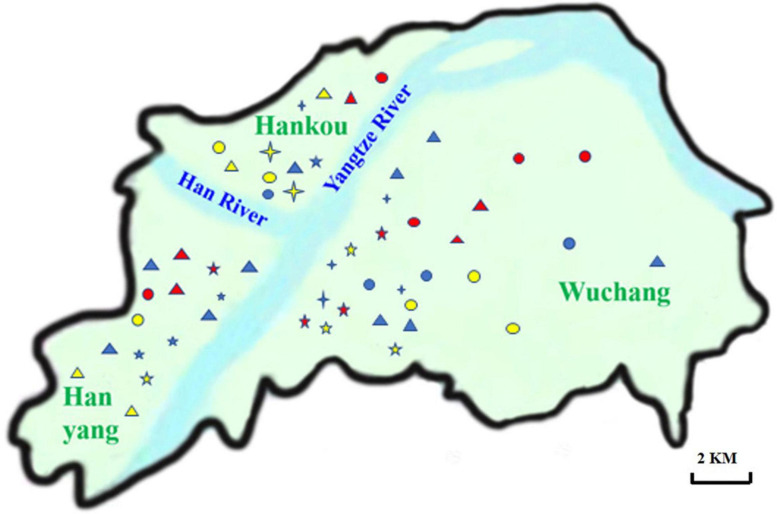
Distribution of supermarket chains in Wuhan. The names of 11 supermarket chains distributed in Hanyang, Hankou and Wuchang were labeled on the Wuhan map. The abbreviations of supermarket chains were listed as follows. 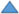, W; 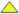, M; 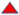, Z; 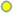, Y; 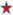, H; 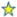, L; 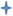, S; 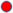, J; 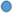, P; 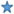, D; 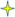, A.

A total of 259 raw pork meat samples were collected from 53 supermarkets in three districts in Wuhan city between January and August in 2019, including Wuchang (*n* = 62), Hankou (*n* = 75), and Hanyang (*n* = 122) (Figure 1). The details of *L. monocytogenes* strains isolated from pork in Wuhan can be found in Table 1. All the samples were collected and transported to the laboratory at 4°C ± 2°C on the same day. Twenty-five-gram pork samples were selectively enriched by UVM modified *Listeria* enrichment broth (Becton, Dickinson and Company, Sparks, MD, United States) at 30°C for 24 h. Then, 100 μL of the UVM culture was added and selectively enriched in 10 mL FB medium (Becton, Dickinson and Company, Sparks, MD, United States) at 37°C. The FB culture was then inoculated and spread on CHROMagar^TM^ plates (Shanghai KeMaJia Biological Technology Co., Ltd., Shanghai, China) at 37°C. The colonies with blue/green sheens and opaque halos on CHROMagar^TM^ plates were inoculated on Bacto^TM^ brain and heart infusion medium (BHI, Becton, Dickinson and Company, Sparks, MD, United States) plates. Typical colonies on BHI plates were identified according to the method reported by Bubert et al. (1999).

**TABLE 1:** Details of *L. monocytogenes* strains isolated from pork in Wuhan.

**Strain**	**Serotype**	**ST**	**Clonal cluster**	**Slaughterhouse**	**Supermarket**	**District**	**Collection time**	**Accession number**
292	1/2c	9	CC9	D	Z1	Hanyang	01-18-2019	ERS5455033
293	1/2a	9	CC9	J	S1	Wuchang	01-18-2019	ERS5455034
295	1/2c	9	CC9	E	Z1	Hanyang	01-18-2019	ERS5455035
296	1/2c	9	CC9	F	Y	Hanyang	01-18-2019	ERS5455036
297	1/2c	9	CC9	F	Y	Hanyang	01-18-2019	ERS5455037
298	1/2a	8	CC8	F	Y	Hanyang	01-18-2019	ERS5455038
299	1/2a	11	CC11	G	Y	Hanyang	01-18-2019	ERS5455039
304	1/2c	9	CC9	G	Y	Hanyang	01-18-2019	ERS5455040
305	1/2a	155	CC155	A	Y	Hanyang	01-18-2019	ERS5455041
306	1/2a	121	CC121	G	W1	Hanyang	01-18-2019	ERS5455042
307	1/2c	9	CC9	G	W1	Hanyang	01-18-2019	ERS5455043
308	1/2c	9	CC9	G	H	Hanyang	01-18-2019	ERS5455044
309	1/2a	155	CC155	G	H	Hanyang	01-18-2019	ERS5455045
310	1/2a	155	CC155	G	H	Hanyang	01-18-2019	ERS5455046
311	1/2a	155	CC155	H	L1	Hanyang	01-18-2019	ERS5455047
312	1/2a	121	CC121	A	W2	Hankou	01-18-2019	ERS5455048
313	1/2c	9	CC9	A	W2	Hankou	01-18-2019	ERS5455049
314	1/2a	204	CC121	B	W2	Hankou	01-18-2019	ERS5455050
315	1/2a	121	CC121	C	M	Hankou	01-18-2019	ERS5455051
316	1/2a	8	CC8	D	L2	Wuchang	01-18-2019	ERS5455052
317	1/2a	155	CC155	A	Z2	Wuchang	01-18-2019	ERS5455053
318	1/2a	155	CC155	I	S1	Wuchang	01-18-2019	ERS5455054
319	1/2a	121	CC121	K	J1	Wuchang	01-18-2019	ERS5455055
320	1/2a	121	CC121	L	P1	Wuchang	01-18-2019	ERS5455056
321	1/2c	9	CC9	A	P1	Wuchang	01-18-2019	ERS5455057
459	1/2c	9	CC9	E	Z3	Hanyang	08-18-2019	ERS5455058
460	1/2c	9	CC9	E	Z3	Hanyang	08-18-2019	ERS5455059
461	1/2a	121	CC121	E	Z3	Hanyang	08-18-2019	ERS5455060
462	1/2a	155	CC155	M	Z3	Hanyang	08-18-2019	ERS5455061
463	1/2a	8	CC8	M	Z3	Hanyang	08-18-2019	ERS5455062
464	1/2a	155	CC155	M	Z3	Hanyang	08-18-2019	ERS5455063
465	1/2a	155	CC155	D	Z3	Hanyang	08-18-2019	ERS5455064
467	1/2a	121	CC121	D	Z3	Hanyang	08-18-2019	ERS5455065
468	1/2a	155	CC155	B	W3	Hanyang	08-18-2019	ERS5455066
469	1/2b	619	CC619	B	W3	Hanyang	08-18-2019	ERS5455067
470	1/2a	155	CC155	B	W3	Hanyang	08-18-2019	ERS5455068
471	1/2b	619	CC619	B	J2	Hanyang	08-18-2019	ERS5455069
472	1/2a	155	CC155	A	J2	Hanyang	08-18-2019	ERS5455070
473	1/2a	155	CC155	A	J2	Hanyang	08-18-2019	ERS5455071
474	1/2a	155	CC155	K	J2	Hanyang	08-18-2019	ERS5455072
475	1/2a	155	CC155	K	J2	Hanyang	08-18-2019	ERS5455073
476	1/2b	619	CC619	N	J2	Hanyang	08-18-2019	ERS5455074
477	1/2b	619	CC619	N	J2	Hanyang	08-18-2019	ERS5455075
478	1/2b	619	CC619	N	J2	Hanyang	08-18-2019	ERS5455076
479	1/2a	155	CC155	G	W4	Hanyang	08-18-2019	ERS5455077
480	1/2a	8	CC8	G	W4	Hanyang	08-18-2019	ERS5455078
481	1/2a	121	CC121	A	W4	Hanyang	08-18-2019	ERS5455079
482	1/2a	204	CC121	A	W4	Hanyang	08-18-2019	ERS5455080
483	1/2a	121	CC121	A	W4	Hanyang	08-18-2019	ERS5455081
484	1/2a	155	CC155	A	W4	Hanyang	08-18-2019	ERS5455082
485	1/2a	8	CC8	F	D	Hankou	08-18-2019	ERS5455083
486	1/2a	155	CC155	F	D	Hankou	08-18-2019	ERS5455084
487	1/2c	9	CC9	A	D	Hankou	08-18-2019	ERS5455085
488	1/2a	155	CC155	I	D	Hankou	08-18-2019	ERS5455086
489	1/2b	2	CC2	I	D	Hankou	08-18-2019	ERS5455087
490	1/2a	155	CC155	B	W5	Hanyang	08-18-2019	ERS5455088
491	1/2a	204	CC121	B	W5	Hanyang	08-18-2019	ERS5455089
492	1/2a	204	CC121	A	W5	Hanyang	08-18-2019	ERS5455090
493	1/2a	155	CC155	A	W5	Hanyang	08-18-2019	ERS5455091
494	1/2a	204	CC121	J	W5	Hanyang	08-18-2019	ERS5455092
495	1/2a	204	CC121	J	W5	Hanyang	08-18-2019	ERS5455093
496	1/2a	204	CC121	G	W5	Hanyang	08-18-2019	ERS5455094
497	1/2a	155	CC155	G	W5	Hanyang	08-18-2019	ERS5455095

### Serotype Identification

The multiplex PCR method was used to detect the PCR serogroups of all isolates according to Doumith et al. (2004). Then, based on PCR serogrouping, antigen serum agglutination and slide agglutination were performed using *Listeria* O and H antisera following manufacturer’s instructions (Denka Seiken Co. Ltd., Tokyo, Japan) to determine the serotypes of isolates.

### Genome Sequencing

The genomes of the isolates were extracted using the TaKaRa MiniBEST Bacteria Genomic DNA Extraction Kit Ver.3.0 (TAKARA, Dalian, China) according to manufacturer’s instructions. Genome sequencing was carried out by the Shanghai Majorbio company. DNA libraries harboring 400 bp DNA fragments were prepared using the NEXTflex^TM^Rapid DNA-Seq following manufacturer’s instructions and pair-end sequenced (2 × 150 bp) on the HiSeq × 10 system (Illumina, San Diego, CA, United States). The quality of the sequence criteria was Q20 ≥ 90%, the depth of sequencing was 100-fold of the genome coverage. The original image data is transferred into sequence data via base calling, which is defined as raw reads and saved as FASTQ file. Quality information by Fastp software V0.20.1^2^ was applied for quality trimming, by which the low-quality data was removed to form clean data. The assembly of the clean reads was performed using SOAPdenovo2^3^. The sequence reads for 64 isolates are deposited and publicly available under the bioproject accession number PRJEB41790. Publicly available genome sequences of 20 clinical isolates were referred to compare the evolutionary relationship (Supplementary Table 2).

### Comparative Genome Analysis

#### MLST and Core Genome MLST (cgMLST)

Sequence types (STs) of the isolates were determined by multilocus sequence typing (MLST), as previously described (Salcedo et al., 2003). The clonal relationships of isolates based on MLST were obtained based on the similarities of the allelic profiles of the seven housekeeping genes to assign the ST as established by the Institut Pasteur MLST^4^ and MLST database^5^ (Ragon et al., 2008).

The cgMLST analysis was carried out using cgMLSTFinder 1.1^6^. The 1,748 core genome multilocus sequence typing (cgMLST) loci used in this study were obtained from BIGSdb-*Lm* platform^7^ (Moura et al., 2016). Phylogenetic analysis of 64 isolates based on 1,748 cgMLST loci was performed using cgmlstfinder.py contained in the cgMLSTFinder service (Clausen et al., 2018). iTOL was used to make the phylogenetic tree visual (Letunic and Bork, 2007).

#### Virulent Gene Typing

The assembled genomes were uploaded to the online analysis software virulence finder 2.0 created by the Center for Genomic Epidemiology (CGE^8^). Pathogenicity island (LIPI)-1, -3, and -4 as well as genes associated with adhesion, invasion, survival, etc., were analyzed with the Database for Virulence Factors of Pathogenic Bacteria (VFDB) (Chen et al., 2005) and ResFinder (Zankari et al., 2012). Virulence genes were considered present when identity was >90% and the coverage was >80%. Genes belonging to LIPI-1, -3, and -4 were detected using BLASTN software. The phylogenetic tree was determined with BioNumerics v7.5 based on the spacer arrangements of virulent genes for each strain.

#### Antibiotic-Resistant Gene Detection

Antibiotic-resistant genes in the isolates were analyzed using the online platform^9^ (Alcock et al., 2020) with the selection criterion as “Perfect and strict hits only,” Nudge ≥95% identity Loose hits to Strict (exclude nudge). The sequences of antibiotic genes and annotations were downloaded, and a maximum likelihood phylogenetic tree was constructed based on the antibiotic profiles using MEGA X software.

#### SNP Typing

Genome sequencing data were obtained by quality filtration and adaptor clearance by fastp. The reads were aligned to reference EGDe using BWA software (Li and Durbin, 2009) and converted to a BAM file. The strains and single nucleotide variations (SNVs) were analyzed with GATK v3.5 (HaplotypeCaller) with the *de novo* algorithm and obtained using the VCF file containing the SNV mutation sites (McKenna et al., 2010). Core single-nucleotide polymorphism (SNP) alignments were used to construct the maximum likelihood phylogenetic tree using SNPhylo pipeline with 1000 bootstraps.

#### CRISPR Typing

CRISPR spacers and repeats were identified using CRISPRFinder^10^ (Grissa et al., 2007). The similarity of each spacer to the sequences deposited in GenBank was analyzed by BLASTN using an e-value cutoff of 0.1. The features of CRISPR in each isolate were input to the binary table, and the maximum likelihood phylogenetic tree was inferred using BioNumerics v7.5 according to the spacer arrangements in each strain.

## Results

### Abundant Diversity of Isolates From Supermarket Chains

**FIGURE 2:**
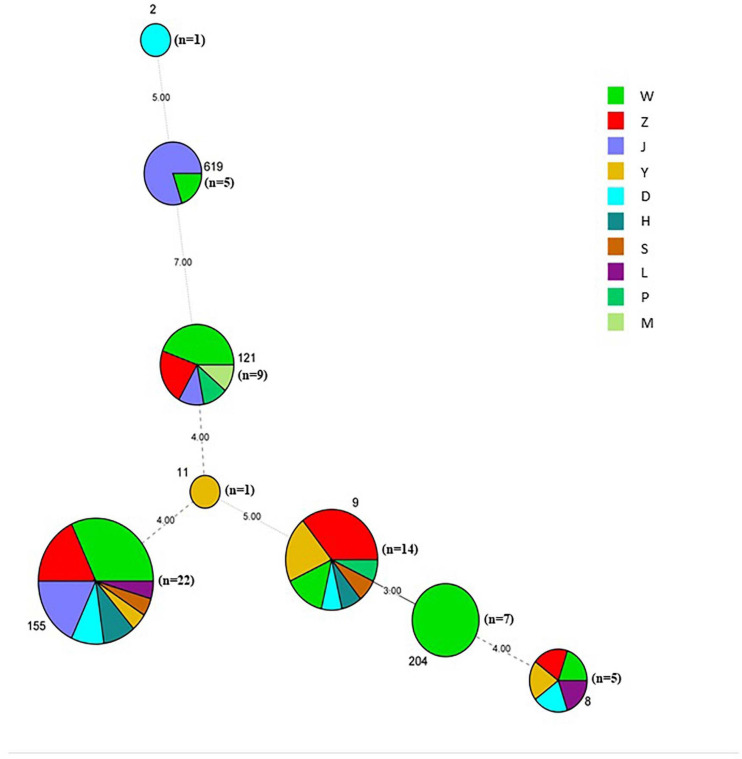
Minimum spanning tree of MLST data for 64 *L. monocytogenes* isolates. Each circle represents one ST, the size of which is related to the number of strains within the ST. The branch numbers account for the number of allele differences between the connected STs. The colors of the circles represent the supermarket chains as indicated. The circle size represents number of isolates, ST2 (*n* = 1), ST8 (*n* = 5), ST9 (*n* = 14), ST121 (*n* = 9), ST619 (*n* = 5), ST11 (*n* = 1), ST155 (*n* = 22), and ST204 (*n* = 7).

A total of 64 *L. monocytogenes* strains were isolated from 259 pork samples (24.71%) in 53 supermarkets belonging to 11 supermarket chains, which were supplied from 14 slaughterhouses (Table 1, Supplementary Figures 1A,B, and Supplementary Table 1). The isolation rate of samples collected from Hanyang (39.34%; 48/122) was obviously higher than those from Wuchang (11.29%; 7/62) and Hankou (12.00%; 9/75) (Supplementary Figure 1A). Additionally, the isolation rates of samples collected in August were higher than those for samples collected in January (42.00% vs. 15.38%). For example, these values for W supermarkets were 48.75% vs. 13.51%, Z supermarkets were 40.00% vs. 13.64%, and J supermarkets were 33.30% vs. 4.76% (Table 1). The isolation rate of samples from W supermarkets was higher than other supermarkets (Supplementary Figure 1B), thus it suggested that the pork sold in this supermarket chain was more contaminated. The isolation rates of W supermarkets were highest for either samples collected in January or August (Table 1). 64 isolates comprised serotypes 1/2a, 1/2b, and 1/2c (Supplementary Figure 1C) belonging to the eight STs (2, 8, 9, 11, 121, 155, 204, and 619), the predominant STs were ST155, ST121, and ST9 (Figure 2 and Supplementary Figure 1D).

**FIGURE 3:**
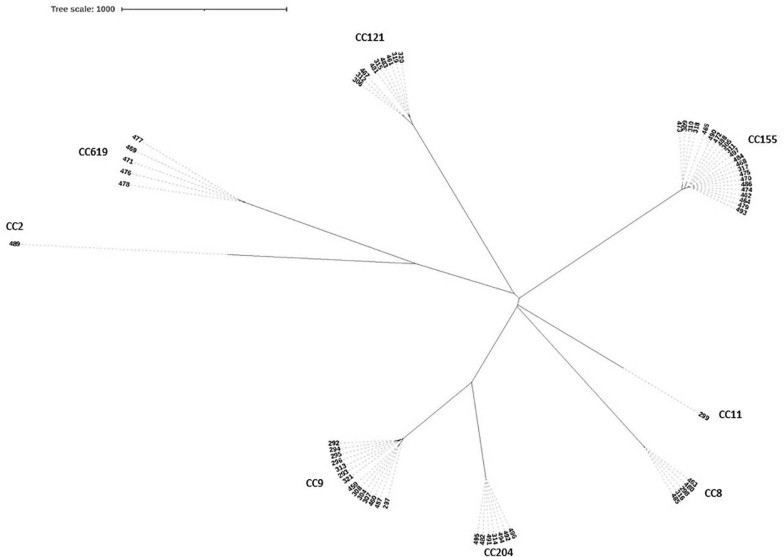
Neighbor-joining tree of *L. monocytogenes* isolates as determined by cgMLST based on the 1,748 core genes. The neighbor joining tree shows the relatedness of the 64 *L. monocytogenes* isolates, each cluster was corresponding to specific CC, the name of per CC was labeled on related cluster.

The high differentiation characterizations of the cgMLST displayed on the neighbor-joining tree (Figure 3) were based on 1,748 core genes. All the obtained isolates were classified into eight CCs, CC8, CC9, CC121, CC155, CC204, CC619, CC2, and CC11. Strikingly, two pairs of CCs, CC2 and CC619 or CC9 and CC204 presented closely phylogenetic relationship. Additionally, three groups in the CC155 cluster and two groups in either CC121 or CC9 cluster clearly demonstrated powerful differentiation characterizations for cgMLST. Interestingly, CC619 strains isolated from supermarket J2 and W3 had closely relationship, which were supplied by the same slaughterhouse B. Isolates Lm485 and Lm298 belonging to CC8 were from supermarket D and Y, respectively, which were supplied from the same slaughterhouse F. Notably, some strains isolated from contaminated pork in different supermarket chains which were supplied from different slaughterhouses were clustered to the same branch. cgMLST analysis can reflect the genetic diversity and relationship, has high discriminatory and traceability, and is a powerful tool to improve surveillance.

### Resistance to Multiple Antibiotic Agents

**FIGURE 4:**
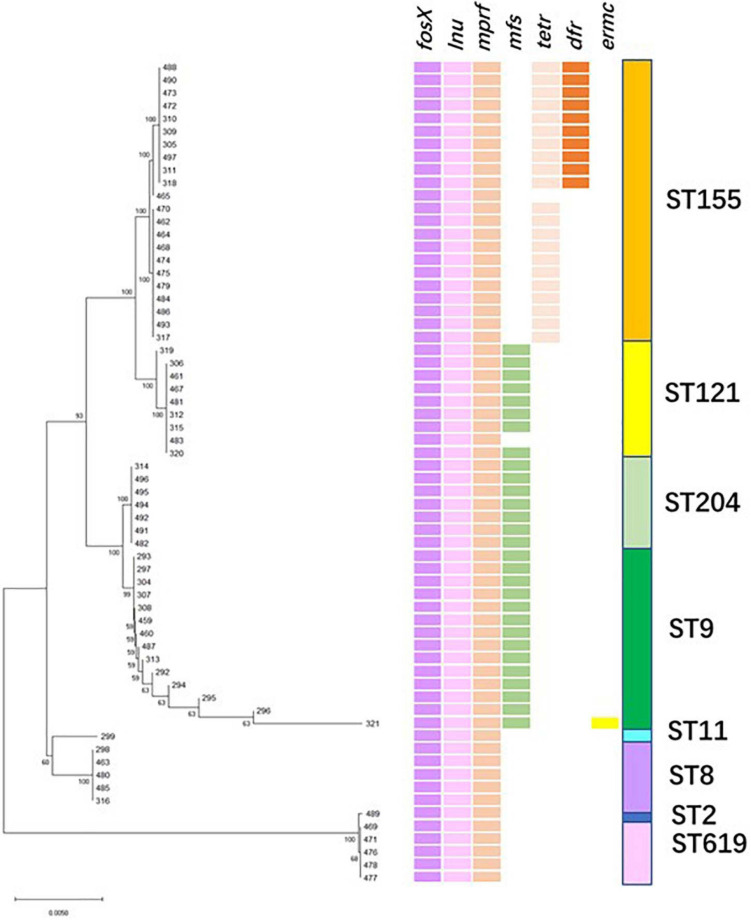
Resistance genes profile. **(Left)** Maximum likelihood phylogenetic tree based on the antibiotic-resistant genes. **(Right)** Patterns of antibiotic-resistant gene presence (colored line) or absence (white). The right column corresponds to the sequence type.

The phylogenetic tree was constructed with sequences of the antibiotic-resistant genes (Figure 4). The entire 64 isolates were grouped into two branches: all 1/2b strains (ST 2, 619) belonged to clade B, and 1/2a and 1/2c strains (ST 8, 9, 11, 121, 155, and 204) were part of clade A. The strains belonging to clade A were further classified into two branches comprising different ST isolates. Interestingly, each ST strain was clustered within the same group and harbored the same antibiotic-resistant genes (Figure 4). Importantly, fosfomycin resistant genes *fosX*, lincosamide resistant gene *lnu*, and multiple peptide resistant gene *mprF*, were present in all the isolates. Additionally, ST204, ST121, and ST9 (except isolate Lm483) strains harbored another resistance gene *mfs*. The ST155 strains were divided into two groups, except for the three antibiotic-resistant genes mentioned above, one group carried the tetracycline-resistance gene (*tetM*), and the other carried *tetM* and gene-encoded dihydrofolate reductase (*dhfR*), our study was the first report on ST155 carrying *dhfR*. Isolates presenting high resistances to fosfomycin have been reported in various countries (Harakeh et al., 2009; Hasegawa et al., 2013). Notably, Scortti et al. (2006, 2018) found that most *L. monocytogenes* strains resisted to fosfomycin *in vitro*–*in vivo* paradox, an epistatic interaction between virulence and resistance genes controlled bacterial susceptibility to fosfomycin *in vivo*. The antimicrobial resistance gene *mprF* that was exclusively present in CC1, CC8, and CC121 was reported in clinical and food isolates (Zuber et al., 2019). Moreno et al. (2014) reported that isolates from slaughterhouse environments, pork and human infections were found to harbor *mprF*. Considering the intricate characteristics of intrinsic and acquired antibiotic resistance, it is significant to determine the antibiotic phenotype and mechanism of these isolates.

### Presence of LIPI-1, LIPI-3, and LIPI-4 in *Listeria* Isolates

**FIGURE 5:**
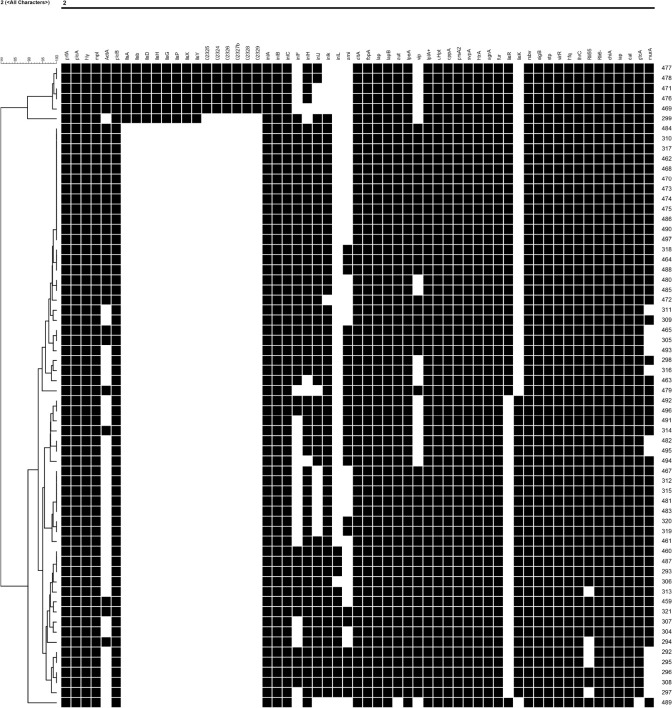
Virulence profiles across the phylogeny of the *L. monocytogenes* isolates. **(Left)** Phylogenetic tree of all isolates determined by 59 virulent and virulence-related genes, part of genes belong. The phylogenic relationship among different STs is shown by the phylogenic tree. **(Right)** Pattern of gene presence (colored line) or absence (white). The presence/absence gene matrix represents, from left to right, genes located in the pathogenicity islands LIPI-1 (*prfA*, *plcA*, *hly*, *mpl*, *actA*, and *plcB*), LIPI-3 (*llsAGHXBYDP*) and LIPI-4 (02325, 02324, 02326, 02328, and 02329), genes coding for internalins (*inlABCFHJKL*) and other genes involved in adherence (*ami*, *dltA*, *fbpA*, *lap*, and *lapB*), invasion (*aut*, *lpeA*, and *vip*), intracellular survival (*hpt*, *lplA1*, *oppA*, *prsA2*, *purQ*, and *svpA*), regulation of transcription and translation (*agrA*, *fur*, *lisR*, *lisK*, *sbV*, *sigB*, *stp*, *virR*, and *hfq*), small non-coding RNA (*lhrC*, *Rli55*, and *Rli6*), cell wall synthesize (*chiA*, *iap*, *dal*, *gtcA*, and *murA*).

In this study, a total of 64 *L. monocytogenes* strains isolated from 11 supermarket chains spanned lineages I and II. The proportions of these isolates within lineages I and II were 90.6 and 9.4%, respectively. LIPI-1 was found in all the isolates, and none of the isolates harbored LIPI-2; LIPI-3 was found in all ST619 and ST2 isolates belonging to lineage II, and LIPI-4 was only distributed in ST619 isolates (Figure 5). Unusually, the ST619 isolates carried three *Listeria* pathogenic islands (LIPI-1, -3, and -4).

To investigate the virulence potentials of the 64 isolates, an *in silico* detection of 91 virulent and virulence-related genes was carried out, main virulent genes in each isolate were presented in Figure 5. Interestingly, either *lisR* or *lisK* was absent in the eight STs, *inlL* was absent in all isolates, *vip* gene was not detected in the ST2, ST8, ST204 and ST619 strains. All ST8, ST11 and ST155 strains carried *inlF* gene. *inlA* encoded truncated InlA presented in 9 ST121 isolates and 3 ST9 isolates. Parts of the ST strains did not contain *ami* and *aut*. Virulent and virulence-associated genes are widely distributed in 64 isolates, particularly the ST619 strains, indicating high virulence features and potential high risk to humans.

### Phylogenetic Relationship of Isolates From Multiple Supply Chains

**FIGURE 6:**
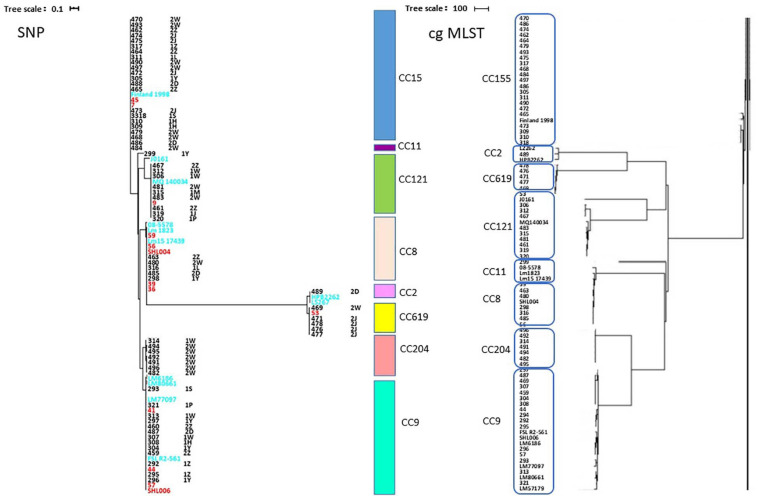
Phylogenetic trees of 64 isolates and 20 clinical isolates constructed from SNPs and cgMLST. **(Left)** SNPs were identified by the SNPhylo pipeline using EDGe as the reference. Strains labeled with light blue color are foreign clinical isolates. Strains labeled with red color are clinical isolates from China. The right column shows the CCs corresponding to the branches of the phylogenetic tree. The words after the names of the isolates are the isolation times and supermarket chains. “1” represents the strains isolated in January 2019. “2” represents the strains isolated in August 2019. The uppercase letters are the abbreviations of the supermarket chains. **(Right)** Neighbor-joining tree of *L. monocytogenes* strains as determined by cgMLST based on the 1,748 core genes. The neighbor-joining tree shows the relatedness of the 64 *L. monocytogenes* isolates and 20 clinical isolates, each blue circle represents one specific CC.

The SNP of each isolate was obtained in comparison with the EGDe strain (Figure 6 and Supplementary Table 3), 8 clusters comprising 8 CCs were consistent with the phylogenetic tree using cgMLST. A part of the isolates from different supermarket chains showed high genetic similarity (0–50 SNP differences). For example, isolates Lm305, Lm317, and Lm484 were isolated from the supermarket chain Y, Z, and W, respectively, however, showed 4 SNP differences between Lm317 and Lm484, and 45 SNP differences between Lm317 and Lm305. Because the pork meats of these 3 supermarket chains were supplied by the same slaughterhouse A, it suggested that the contamination of pork was possibly sourced from the same production chain. Additionally, the isolates were from different supermarket chains and collection times, but the SNP differences were less than two. For example, despite the temporal (half year gap) and geographical differences among the isolates, Lm459, Lm304, Lm487, and Lm307 indicated the persistence of specific *L. monocytogenes* in the pork production chain. 12 strains comprising 5 STs were isolated in pork meat from supermarket chain J, Z, and W, which were supplied by the same slaughterhouse E; the pigs transported to the slaughterhouse E were from four different pig farms (HP, SP, GS3, and YJ). Therefore, various diversity of the strains was possibly due to the intricate supply chain. Importantly, specific ST isolates such as ST8 showed high genetic similarity (<21 SNP differences) to clinical isolates Lm 1823 and SHL004 (Supplementary Table 2). This represented a public health concern because of their potential to transfer to humans via the food chain.

### CRISPR-Cas System-Based Subtyping Contributing to Improve the Discrimination

**FIGURE 7:**
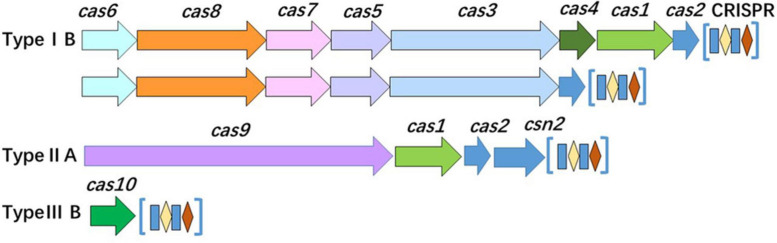
Organization of the CRISPR-Cas system in the isolates. The CRISPR-Cas loci of class 1 (types IB and IIIB) and class 2 (type IIA) are presented.

Analysis of the CRISPR-Cas system of isolates revealed that the system was distributed in most of the isolates (37/64, 57.81%). Based on the number and diversity of the CRISPR-Cas system, these isolates were divided into two classes (classes 1 and 2) and three types (types IB, IIA, and IIIB) (Figure 7 and Table 2). Among 8 STs, only ST121, ST155, and ST619 isolates harbored complete CRISPR-Cas system. Interestingly, all of the ST121 strains carried both types IB and IIA CRISPR-Cas systems (Figure 7 and Table 2), and CRISPR loci of ST155 isolates existed in both upstream and downstream of Cas proteins. Other STs only carried Cas10 protein or one to four Cas3 proteins, particularly of carrying limited spacers (between 0 and 10) (Table 2). The ST9 isolates carried either Cas10 or Cas3 proteins (Table 2). As mentioned above, the genotypes of the CRISPR-Cas system were observed to be correlated with the STs.

**TABLE 2:** Organization of Cas proteins and CRISPRs.

**Types**	**Cas proteins**	**STs**	**Number of Spacers**	**Isolates**
Type Ib	Cas6, Cas8, Cas7, Cas5, Cas3, Cas4, Cas1 (1026 bp), Cas2	ST155	10 + 58	317, 462, 470, 474, 475, 484, 486
			16 + 157	493
			10 + 57	470
	Cas6, Cas8, Cas7, Cas5, Cas3, Cas4, Cas1 (999 bp), Cas2	ST155	10 + 58	309, 310, 311, 472
			10 + 57	318
			10 + 61	305
			10 + 67	488
			22 + 156	465
			22 + 143	464
		ST619	28	471, 478, 476
			49	477
			78	469
	Cas6, Cas8, Cas7, Cas5, Cas3, Cas2	ST121	5 + 35	306, 315, 483, 312, 461, 481, 467
			46	320
			35	319
Type IIa	Cas9, Cas1, Cas2, Csn2	ST121	423	306, 315, 483
			27	312
			40	461
			44	481
			47	467
			22	320
			21	319
Type IIIb	Cas10	ST9	4 + 1	313, 297, 307, 487
			4 + 1 + 1	321
			4 + 1 + 1 + 1 + 1	459
Cas3	Cas3^#^ Cas3^#^Cas3^#^Cas3^#^	ST8	1 + 7 + 2 + 0	316
	Cas3^φ^Cas3*Cas3*Cas3*	ST8	1 + 7 + 0 + 0	298
	Cas3^#^ Cas3^#^Cas3^#^	ST8	1 + 7 + 1	480, 485
	Cas3^#^ Cas3^#^Cas3^#^	ST8	9 + 1 + 0	463
	Cas3^#^ Cas3^#^Cas3^#^	ST2	0 + 2 + 4 + 1	489
	Cas3*Cas3*Cas3*	ST9	4 + 1 + 1	293, 294
	Cas3*Cas3*Cas3*	ST9	6 + 1 + 0	296
	Cas3^φ^Cas3^#^	ST9	4 + 0	295
	Cas3*Cas3*	ST9	4 + 0	292
	Cas3^#^Cas3^#^	ST204	2 + 0	482
	Cas3^#^Cas3^#^	ST204	1 + 0	491
	Cas3*Cas3*	ST204	2 + 0	495
	Cas3*Cas3*	ST11	7 + 0	299
	Cas3^#^	ST204	1	314
	Cas3^#^	ST204	0	494, 496
	Cas3*	ST9, ST204	0	304, 492

*“+” means that there are two separate CRISPR loci in the genome.*

Cas3*, Cas3 (3,540 bp); Cas3^#^, Cas3 (3,573 bp); Cas3^φ^, Cas3 (1,569 bp).

It is worth noting that there was variant organization of CRISPRs in the same ST (Table 2 and Supplementary Figure 2), for example, either of type IIA CRISPR-Cas system of ST121 isolates or types IB CRISPR-Cas system of ST155 isolates comprised 7 kinds of CRISPRs. The diversity of the CRISPR-Cas system indicates the role of various functions in the corresponding strains, which are possibly related to different STs encountered in distinct environments. Interestingly, 13 pairs of the isolates (0 SNP difference) were indistinguishable by SNP subtyping, but these isolates could be differentiated adequately by CRISPR-Cas subtyping. For example, three 0-SNP-difference strains isolated from the same supermarket chain were found to harbor various CRISPR spacers. In brief, these results verify that the organization of the CRISPR-Cas system exhibits genetic diversity of isolates, thereby reflecting the higher discrimination ability.

## Discussion

*Listeria monocytogenes* is a severe foodborne pathogen and is a significant threat to public health. Animal-derived *Listeria*-contaminated food is generally understood to be the main cause of listeriosis outbreaks (de Valk et al., 2001; Choi et al., 2014), this pathogen with the evidence of zoonotic transmission causes global economic and health burdens (Halbedel et al., 2020; Thomas et al., 2020). Therefore, improving the detection of bacterial contamination of pork, beef and poultry is an efficient measure to prevent human listeriosis.

Genome sequencing has been gradually used to reveal the detailed characteristics of typing, virulent, and antibiotic-resistant genes and has become a useful investigation tool to support public health (Halbedel et al., 2018; Allard et al., 2019). The cgMLST and SNP have the advantage of high discriminatory power and are widely used for surveillance by appropriate public health analysts and epidemiologists (Hurley et al., 2019; Jagadeesan et al., 2019; Rivas et al., 2019; Papić et al., 2020), whereas CRISPR subtyping is rarely used for *Listeria* molecular epidemic investigations. In the present study, based on isolates from raw pork meat, WGS was applied to obtain information on cgMLST, SNP, and the CRISPR-Cas system. The same clusters were classified by either cgMLST or SNP. It was observed that isolates from clinical sporadic and outbreak cases could be clustered together with the pork isolates obtained in this study as they had close relationships, which was reflected in the SNP differences. Furthermore, they indicated that pork isolates have a potentially high risk of causing human listeriosis along the food chain.

CRISPR-Cas systems are adaptive immunity systems in many prokaryotic microorganisms; they confer defense capabilities against horizontal gene transfer elements and display variations in different species (Marraffini, 2015; Koonin et al., 2017). CRISPR-based genotyping applications have been exploited for some species, which have been classified into 2 classes, 6 types, and 33 subtypes (Makarova et al., 2020). In the case of *Salmonella enterica* serovar Typhimurium, the profile of spacer arrangements has strong association with strain phylogeny (Xie et al., 2019). However, its application to *L. monocytogenes* is limited owing to difficulties in acquiring CRISPR structures from the *Listeria* genotype, particularly in the lineage I strains (Di et al., 2014). *RliB*-CRISPR is ubiquitously distributed among *Listeria* genome (Sesto et al., 2014). Taylor and Stasiewicz (2019) indicated that CRISPR subtyping cannot improve differentiation of persistent and sporadic *L. monocytogenes* strains. By combining the organization of Cas proteins and the arrangement of CRISPR loci of isolates, we found that types IB, IIA, and IIIB displayed their unique variations in the Cas proteins and spacers, and there were remarkable features of Cas3 proteins and CRISPRs in other strains. Importantly, *L. monocytogenes* strains with closely evolutionary relationship were indistinguishable by SNP subtyping, but they could be differentiated adequately by CRISPR subtyping. Our results indicated that combining the organization of Cas proteins and CRISPR loci could be a complementary measure to discriminate genetically close “indistinguishable” strains harboring CRISPR. CRISPR-Cas system plays multiple roles beyond adaptive immunity, i.e., gene regulation and virulence (Faure et al., 2019). Understanding the structure and function of CRISPR-Cas system contributes to develop useful technology and products to control pathogen, i.e., phages of *Listeria* offer novel tools for detection, differentiation, CRISPR-Cas-assisted phage engineering, diagnostics, and biocontrol (Hagens and Loessner, 2014; Hupfeld et al., 2018; Meile et al., 2020).

*Listeria monocytogenes* carrying different virulence factors are highly heterogeneous distributions between CCs and lineages (Tavares et al., 2020). The four known *Listeria* pathogenicity islands play vital roles in *L. monocytogenes* survival and infection in hosts, which were mainly described for lineage I (LIPI-1, -3, and -4) and sublineage II (LIPI-1, and LIPI-2) (Vázquez-Boland et al., 2001; Cotter et al., 2008; Maury et al., 2016; Yin et al., 2019). Maury et al. (2016) found that CC1, CC6, CC4, and CC2 belonging to Lineage I are the dominant CCs causing listeriosis in Western countries, particularly, CC4 carrying both LIPI-1 and 4 confers hypervirulence by enhancing invasion into the CNS and placenta. Whereas, sporadic listeriosis is the primary feature of clinical cases recorded in China, the CCs -87, -8, -9, -121, -155, and -619 were present in human clinical cases (Li et al., 2019; Zhang et al., 2019a; Yin et al., 2020), CC87 isolates harboring LIPI-1, -3, and -4 were one of the most prevalent hypervirulent CC in clinical isolates (Li et al., 2019; Zhang et al., 2019a; Yin et al., 2020). In this study, none of CC87 strains were isolated, while a unique clonal complex CC619 isolates carrying LIPI-1, -3, and -4 belonging to lineage II were isolated from different supermarket chains, which have closely evolutionary relationship with clinical ST619 isolates (50 SNP differences), suggesting that they have high risk to cause listeriosis along food production chain. Thus, the CC isolates observed in this study implied that although the occurrence of putatively hypervirulent isolates was not high, all isolates associated with the pork production chain could have substantial public health implications.

In this study, WGS was applied as an investigation tool to detect the status of *L. monocytogenes* contamination via raw pork sold in supermarkets. Specific CRISPR-Cas systems contribute to improve the discrimination of genetically close “indistinguishable” strains. Some supermarket chains have different supply channels which results in the diversity of isolates. Multiple farms supplying pigs to the same slaughterhouse possibly caused the abundant diversity of the strains isolated from same supermarket chains. Importantly, closely evolutionary relationship exists in isolates from the pork sold in different supermarket chains and supplied from the same slaughterhouse, which indicates that *L. monocytogenes* spread along the slaughter production chain. The genetic similarities between isolates from different supermarket chains suggest the importance and necessity of tracking and detecting the sources of meat supply at both the slaughterhouse and farm levels. The high isolation rates from contaminated pork meat supplied via 11 supermarket chains in Wuhan city signify the urgent need to strengthen pathogen surveillance systems.

## Data Availability Statement

The data presented in this study are deposited in the European Nucleotide Archive repository, accession number PRJEB41790.

## Author Contributions

ML and YW designed the experiments. YW and QJ performed experiments and analyzed the results. SL guided the data analysis. YW and ML wrote the manuscript. All authors read and approved the final manuscript.

## Conflict of Interest

The authors declare that the research was conducted in the absence of any commercial or financial relationships that could be construed as a potential conflict of interest.

## Footnotes

1 https://www.cdc.gov/listeria/outbreaks/index.html

2 https://github.com/OpenGene/fastp

3 https://github.com/aquaskyline/SOAPdenovo2

4 http://www.pasteur.fr/mlst

5 https://cge.cbs.dtu.dk/services/MLST/

6 https://cge.cbs.dtu.dk/services/cgMLSTFinder/

7 http://bigsdb.pasteur.fr/listeria

8 https://cge.cbs.dtu.dk/services/VirulenceFinder/

9 https://card.mcmaster.ca/analyze/rgi

10 https://crisprcas.i2bc.paris-saclay.fr/CrisprCasFinder/Index

## Supplementary Material

Supplementary Figure 1 Numerical representation of the 64 isolates. **(A)** Isolation rate of *L. monocytogenes* from pork meat in 3 districts. **(B)** Isolation rate of *L. monocytogenes* from pork meat in 11 supermarket chains. **(C)** Ratio of serotypes among strains isolated in 11 supermarket chains. **(D)** Distribution of sequence type.

Supplementary Figure 2 Cluster analysis of CRISPR loci in 64 isolates.

Supplementary Table 1 Farms for E and G slaughterhouses.

Supplementary Table 2 Information of clinical isolates.

Supplementary Table 3 SNP analysis of pork isolates, international reference strain and clinical isolates.
