# Supplementary figures and images for: Prevalence and Genetic Diversity of Listeria monocytogenes Isolated From Retail Pork in Wuhan, China
Source: Front Microbiol. 2021 Mar 9;12:620482. doi: 10.3389/fmicb.2021.620482 (PMC7986423; doi:10.3389/fmicb.2021.620482)

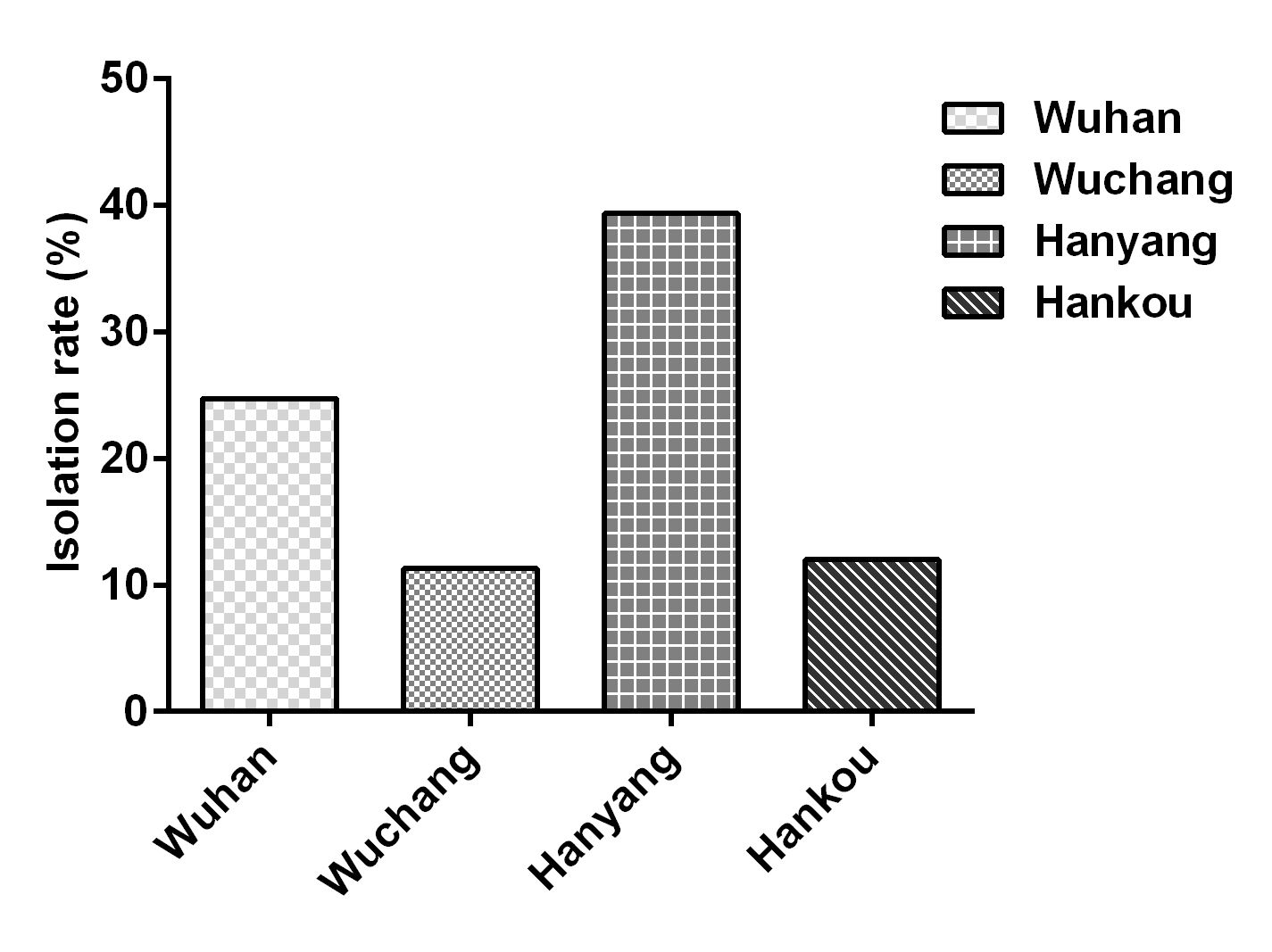

Supplement: Supplementary Figure 1 — Numerical representation of the 64 isolates. (A) Isolation rate of L. monocytogenes from pork meat in 3 districts. (B) Isolation rate of L. monocytogenes from pork meat in 11 supermarket chains. (C) Ratio of serotypes among strains isolated in 11 supermarket chains. (D) Distribution of sequence type. [file Image_1.JPEG]

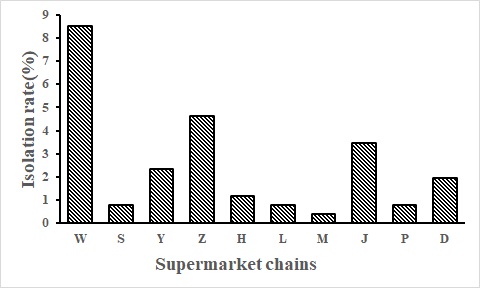

Supplement: Supplementary Figure 2 — Cluster analysis of CRISPR loci in 64 isolates. [file Image_2.JPEG]

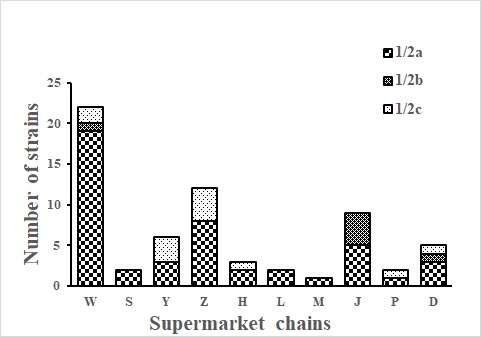

Supplement: Supplementary file 3 [file Image_3.JPEG]

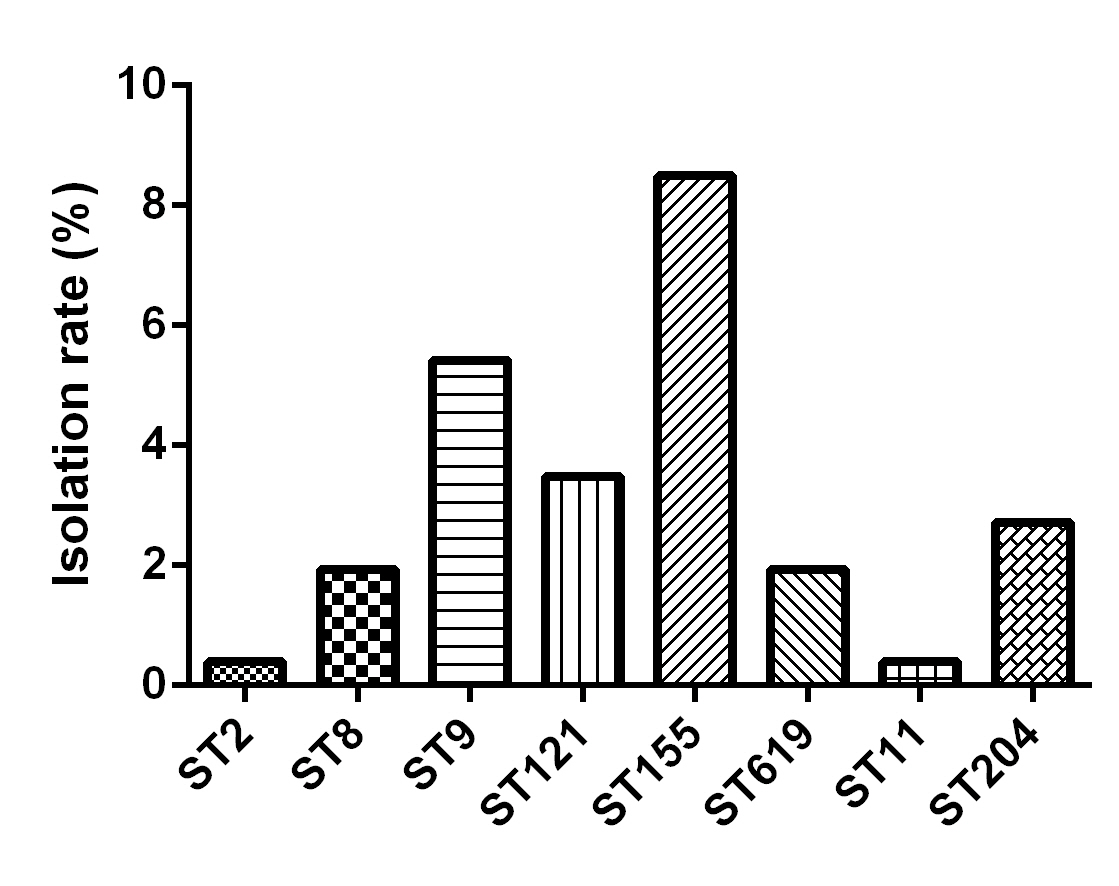

Supplement: Supplementary file 4 [file Image_4.JPEG]

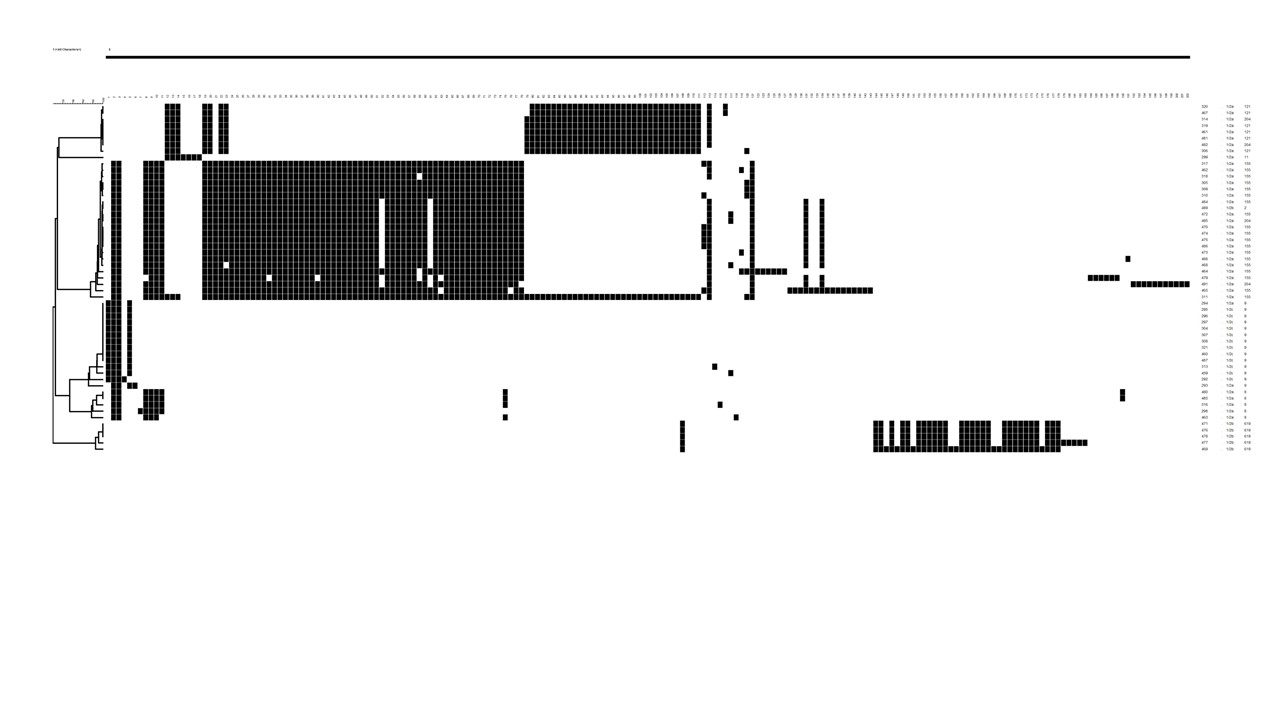

Supplement: Supplementary file 5 [file Image_5.JPEG]
